# Efficient Gradient Updating Strategies with Adaptive Power Allocation for Federated Learning over Wireless Backhaul

**DOI:** 10.3390/s21206791

**Published:** 2021-10-13

**Authors:** Yunji Yang, Yonggi Hong, Jaehyun Park

**Affiliations:** Division of Smart Robot Convergence and Application Engineering, Department of Electronic Engineering, Pukyong National University, Busan 48513, Korea; yyj26@pukyong.ac.kr (Y.Y.); yongki0503@hanmail.net (Y.H.)

**Keywords:** federated learning, image classification, wireless backhaul, aggregated gradient updating

## Abstract

In this paper, efficient gradient updating strategies are developed for the federated learning when distributed clients are connected to the server via a wireless backhaul link. Specifically, a common convolutional neural network (CNN) module is shared for all the distributed clients and it is trained through the federated learning over wireless backhaul connected to the main server. However, during the training phase, local gradients need to be transferred from multiple clients to the server over wireless backhaul link and can be distorted due to wireless channel fading. To overcome it, an efficient gradient updating method is proposed, in which the gradients are combined such that the effective SNR is maximized at the server. In addition, when the backhaul links for all clients have small channel gain simultaneously, the server may have severely distorted gradient vectors. Accordingly, we also propose a binary gradient updating strategy based on thresholding in which the round associated with all channels having small channel gains is excluded from federated learning. Because each client has limited transmission power, it is effective to allocate more power on the channel slots carrying specific important information, rather than allocating power equally to all channel resources (equivalently, slots). Accordingly, we also propose an adaptive power allocation method, in which each client allocates its transmit power proportionally to the magnitude of the gradient information. This is because, when training a deep learning model, the gradient elements with large values imply the large change of weight to decrease the loss function.

## 1. Introduction

Recently, deep neural networks (DNNs) or convolutional neural networks (CNNs) have been widely applied to complicated signal processing, such as classification tasks and signal regression problems, due to their outstanding performances in nonlinear adaptability and feature extraction ([1,2,3] and references therein) and are also extended to the distributed sensing systems (e.g., the object recognition using distributed micro-Doppler radars in [4] and the data driven digital healthcare applications [5,6,7]). In the distributed sensing systems, centralized training strategies may be adopted to train their common DNN or CNN modules by sharing their sensing data. However, due to the data-size and the privacy issues of the locally collected data, the centralized training is not desirable, especially when the capacity of the backhaul link for the data exchange is limited.

The federated learning approach has been extensively investigated as an alternative distributed machine learning method [8,9] where, rather than sharing their locally collected dataset, the clients report the stochastic gradient information (minimizing the loss function with respect to their local dataset) to the main server. The main server then aggregates the stochastic gradient information and broadcast it to the clients. Accordingly, to achieve the unbiased stochastic gradient at the main server, the training data sampling methods are investigated [10,11]. Furthermore, in [12], to reduce the communication overhead of transmitting the updated gradient information (proportional to the number of weights in the DNNs and CNNs), an efficient weight aggregation protocol for federated learning is proposed and in [13], the structured updating method is proposed for the communication cost reduction. However, they assume that the stochastic gradient information is perfectly transferred from the multiple clients to the main server without any distortion.

In the federated learning process, when the clients are connected with wireless-connected clients, local gradient information needs to be transferred from the distributed clients to the server over the wireless backhaul link and can be distorted due to wireless channel fading. In [14,15,16,17], for the wireless backhaul, the federated learning strategies are proposed for the MNIST hand-writing image classification and the associated wireless resources are efficiently optimized. In [14,15], the average of the local stochastic gradient vectors is recovered at the server when the pre-processed local gradient vectors are transferred from the clients. In [16], the compressive sensing approach is proposed to estimate the local gradient vectors at the server. In [17], joint communication and federated learning model is developed, where the resource allocation and the client selection methods are proposed such that the packet error rates of the communication links between server and clients are optimized. We note that most of the previous works have focused on the estimation of local stochastic gradient vectors at the server.

In this paper, we also consider the federated learning system, where distributed clients are connected to the server via wireless backhaul link and develop efficient training strategies for the federated learning over wireless backhaul link. Differently from the previous works, where the average of the local stochastic gradient vectors (i.e., the equal-weight combining) is recovered at the server, we propose an efficient gradient updating method, in which the local gradients are combined such that the effective signal-to-noise ratio (SNR) is maximized at the server. In addition, we also propose a binary gradient updating strategy based on thresholding in which the round associated with all channel having small channel gains is excluded from federated learning. That is, when the backhaul links for all clients have channel gain smaller than a pre-defined threshold simultaneously, the server may have severely distorted gradient vectors, which can be avoided through the proposed updating with thresholding. Furthermore, because each client has limited transmission power, it is effective to allocate more power on the channel slots carrying specific important information, rather than allocating power equally to all channel resources (equivalently, slots). Accordingly, we also propose an adaptive power allocation method, in which each client allocates its transmit power proportionally to the magnitude of the gradient information. This is because, when training a deep learning model, the gradient elements with large values imply the large change of weight to decrease the loss function.

Through the extensive computer simulations, it can be found that the proposed gradient updating methods improve the federated learning performance over the wireless channel. Specifically, due to the distortion over wireless channel, the classification accuracy of the equal-weight combining decreases drastically as the rounds of the federated learning increase. In contrast, the proposed effective SNR maximizing scheme with thresholding exhibits the accuracy performance which is comparable to that for the federated learning over the error-free backhaul link. We note that, as the threshold level increases, the federated learning is performed stably, because the highly distorted gradient update vector due to small channel gain can be discarded by a large threshold level. However, the large threshold level may incur the gradient updating delay, but the adaptive power allocation strategy can improve the trade-off between the federated learning performance and the learning delay due to the threshold level.

The rest of this paper is organized as follows. In Section 2, the system model for the federated learning system with the wireless backhaul is presented in which the distributed clients have a common CNN module for the handwriting character recognition. In Section 4, gradient updating methods are proposed. In addition, the adaptive power allocation method is also developed considering the importance of the gradient information. In Section 5, we provide several simulation results and in Section 6, we give our conclusions.

## 2. System Model

**Figure 1:**
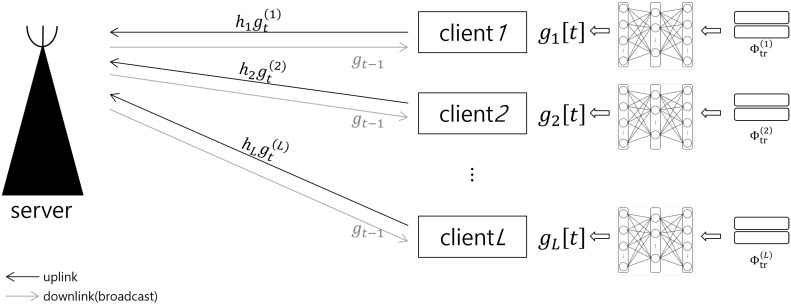
Federated Learning over wireless communication system for target classification.

In Figure 1, we consider the federated learning systems with wireless backhaul, where the *L* multi-clients have their own datasets to train each local network. Here, a common neural network model is shared for all clients and it is trained through the federated learning over wireless backhaul connected to the main server. The common neural network is designed for the classification problem, in which the label $\hat{d}$ is induced from the network output for the *l*th client’s measured data with the label *d*, $S_{d}^{\left(l\right)}$. That is,

(1) $$\begin{array}{ccc} \; & \hat{d}\;=\;\underset{d=1,\ldots ,D}{\arg\max}\;x_{out}\left[d\right], & \\ \; & s.t.\;x_{out}\;=\;f\left(S_{d}^{\left(l\right)};\theta \right)\in R^{D\times 1} & \end{array}$$

where f(;θ) denotes the non-linear neural network function with the model parameter $\theta (\in R^{P\times 1})$ that gives the estimate of the categorical label probability vector as its output vector. Here, *P* denotes the number of weights in the common neural network model and $x_{out}\left[d\right]$ is the *d*th element of the vector $x_{out}$. We note that the size of the model parameter (*P*) is determined by the structure of the neural network model. Specifically, in the case of a convolutional layer with *K*$K_{f1}\times K_{f2}$ filters, the number of weights is given as $K_{f1}\times K_{f2}\times K+K$ that accounts for the kernel size ($K_{f1}\times K_{f2}$), the number of kernels (*K*) and the number of biases (*K*). In the case of a single fully-connected layer, the number of weights is calculated as $N_{in}\times N_{nr}+N_{nr}$, where $N_{in}$ and $N_{nr}$ denote the input size and the number of neurons, respectively. See also Section 2.1. We note that, because the collected data at each client are generally of a large dimension with private security issues, it is not desirable to report the collected data to the server. Furthermore, the large dimension of the data may cause the significant burden on the typical backhaul link to transmit a number of training datasets. Instead, the neural network model f(;θ) will be shared over all clients and θ can be locally trained with the data obtained from each client. By denoting $\theta^{\left(l\right)}$ as the model parameter trained at the *l*th client, $\theta^{\left(l\right)}$ is reported to the server through the wireless uplink backhaul for the federated learning. The associated federated learning strategies and power allocation over the wireless backhaul will be discussed in more detail in Section 4.

### 2.1. CNN Architecture for Handwriting Character Recognition

**Figure 2:**
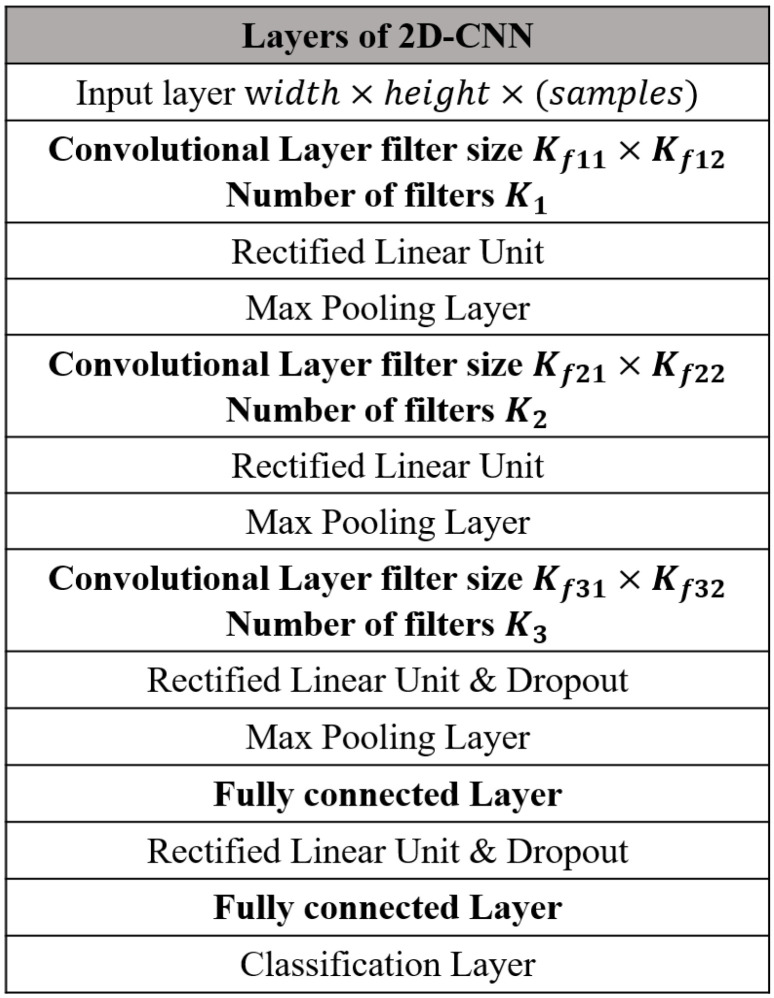
CNN module for handwriting character recognition.

Throughout the paper, multiple clients have a common neural network for the handwriting character recognition. Specifically, a typical CNN module is considered for the character image classification as in Figure 2, but the proposed federated learning strategy can be applied to other CNN models. The non-linear neural network function $f(S_{i}^{\left(l\right)};\theta )$ in (1) is composed of an input layer, convolutional layers, activation layers, max pooling layer, a fully-connected layer, and an output layer. See Section 4 for the specific values of the hyperparameters of CNN module.

*– Convolution Layer*: The handwriting image matrix, $S_{i}^{\left(l\right)}\in R^{N_{width}\times N_{height}}$ is exploited as the input of the convolution layers. In addition, each element of their output is computed through the convolution operation with a $K_{fi1}\times K_{fi2}$ filter (equivalently, kernel) for *i*th layer. Specifically, the output of the *i*th convolution layer can be given as:

$$\begin{array}{c} \;\;X_{\left(i\right)}\left[m,n,k\right]=\;\;\;\;\;\;\;\;\;\;\;\;\;\;\;\;\;\;\;\\ \sum_{p\;=\;0}^{K_{fi1}\;-\;1}\;\sum_{q\;=\;0}^{K_{fi2}\;-\;1}\;\sum_{k\;=\;0}^{K_{i}\;-\;1}\;f_{a}\;\left(\;X_{\left(i\;-\;1\right)}\left[m\;+\;p,n\;+\;q,k\right]W_{\left(i\right)}\left[p,q,k\right]\;\right)\;+b_{\left(i\right)}\left[k\right], \end{array}$$

where $X_{\left(i-1\right)}\left[m,n,k\right]$ is the (m,n,k)th element of $X_{\left(i-1\right)}\in R^{m_{i-1}\times n_{i-1}\times k_{i-1}}$, the input of the *i*th layer and $f_{a}\left(\cdot \right)$ is an activation function. In addition, $W_{\left(i\right)}\left[p,q,k\right]$ is the (p,q,k)th element of the filter matrix $W_{\left(i\right)}$ at the *i*th layer and $b_{\left(i\right)}\left[k\right]$ is the *k*th element of a bias vector $b_{\left(i\right)}$. Throughout the paper, rectified linear unit (ReLU) function is used as the activation function, which is given as

$$f_{a}\left(X_{\left(i\right)}\right)=max\left(0,X_{\left(i\right)}\right).$$

*– Max pooling layer:* In the pooling layer, to reduce the dimension of the input data without losing useful information, the elements of the input are down-sampled [18]. In the Max pooling layer, after dividing the input matrix into multiple blocks, the maximum value in each block is sampled and forwarded to the dimension-reduced output matrix.

*– Flatten, Fully-Connected (FC) layer:* The flatten layer is used for changing the shape of output of convolution layer into the vector which is used as the input of FC layer. We note that, in the case of a single fully-connected layer with $N_{in}$ input elements and $N_{nr}$ neurons, the number of weights is given as $N_{in}\times N_{nr}+N_{nr}$. In the FC layer, the output of convolution layer is associated with a proper loss function such that the label is correctly identified after the training.

Throughout the paper, the cross entropy (CE) is used as the loss function which is given as

(2) $$\begin{array}{ccc} \;L_{CE}\left(x_{out},{\bar{L}}_{d};\theta \right)\; & \;=\; & \;-\sum_{i=1}^{D}{\bar{L}}_{d}\left[i\right]\log\left(x_{out}\left[i\right]\right), \end{array}$$

where $x_{out}\in R^{D\times 1}$ is the output of FC and ${\bar{L}}_{d}$ is a label one-hot encoded vector of size *D* that has zeros in all elements except the *d*th element, which is assigned a value of 1. Then, by using the local training datasets ($\Phi_{tr}^{\left(l\right)}={\left\{S_{d,tr}^{\left(l\right)},{\bar{L}}_{d,tr}\right\}}_{t=1}^{N_{tr}}$) at the *l*th client, the network function parameter can be updated as:

(3) $$\begin{array}{c} \theta_{t}^{\left(l\right)}\leftarrow \theta_{t-1}^{\left(l\right)}+g_{t-1}^{\left(l\right)}, \end{array}$$

where $g_{t-1}^{\left(l\right)}\left(\in R^{P\times 1}\right)$ denotes the gradient such that the loss function is minimized for the local training datasets $\Phi_{tr}^{\left(l\right)}$ and is given as $g_{t-1}^{\left(l\right)}≜-{\left.\eta {▽}_{\theta }\;L_{CE}\left(x_{out},{\bar{L}}_{d,tr};\theta \right)\right|}_{\theta =\theta_{t-1}^{\left(l\right)}}$ with a learning rate, η.

### 2.2. Signal Model for Wireless Backhaul

As in Figure 1, the clients are connected to the server through the wireless backhaul link. For the federated learning, the model parameters aggregated at the server are broadcast at each iteration of training phase through the wireless downlink channel, while the model parameters trained at the *l*th client are reported to the server through the wireless uplink backhaul link. Throughout the paper, we focus only on the uplink phase of multiple access channel and assume that the broadcast channel for the downlink phase is error-free, as done in [15,16,19].

Assuming that the clients and the server have a single antenna for the backhaul link, when total *B* channel resources with narrowband signal bandwidth are available (Here, we note that the channel resources may be given in the frequency axis or may be given in the time axis.), the received signal at server for the *t*th round of the gradient update can be given as

(4) $$y_{t}\left[b\right]=\sum_{l=1}^{L}h_{l,t}\left[b\right]x_{l,t}\left[b\right]+n_{t}\left[b\right],$$

for b=1,…,B, where $x_{l,t}\left[b\right]$ is the precoded transmit signal of the *l*th client at the *b*th channel resource with $E[|x_{l,t}\left[b\right]{|}^{2}]=1$ for the *t*th round. Here, $h_{l,t}\left[b\right]$ and $n_{t}\left[b\right]$ denote the aggregated Rayleigh fading channel and the zero-mean additive white Gaussian noise (AWGN) at the *b*th channel resource, respectively. That is, $h_{l,t}\left[b\right]$ follows a Gaussian distribution with a zero-mean and a variance $\sigma_{h_{l}}^{2}$ (that is, $h_{l,t}\left[b\right]∼N\left(0,\sigma_{h_{l}}^{2}\right)$). Likewise, $n_{t}\left[b\right]∼N\left(0,\sigma_{n}^{2}\right)$. In addition, the wireless channel is constant over each round of federated learning process, but changes independently from round to round. By concatenating $y_{t}\left[b\right]$ in (4), the received signal at server can be vectorized as:

(5) $$y_{t}=\left[\begin{array}{c} y_{t}\left[1\right]\\ \vdots \\ y_{t}\left[B\right] \end{array}\right]=\sum_{l=1}^{L}H_{l,t}x_{l,t}+n_{t},$$

where $H_{l,t}=diag\left\{h_{l,t}\left[1\right],\ldots ,h_{l,t}\left[B\right]\right\}$ and

$$x_{l,t}=\left[\begin{array}{c} x_{l,t}\left[1\right]\\ \vdots \\ x_{l,t}\left[B\right] \end{array}\right],\;n_{t}=\left[\begin{array}{c} n_{t}\left[1\right]\\ \vdots \\ n_{t}\left[B\right] \end{array}\right].$$

Here, $diag\left\{a_{1},\ldots ,a_{B}\right\}$ denotes a B×B diagonal matrix having its diagonal elements as $a_{1},\ldots ,a_{B}$.

## 3. Federated Learning for Handwriting Character Recognition

Note that, as in (3), the CNN parameter $\theta^{\left(l\right)}$ can be trained with the local training datasets at each client, which limits the adaptability of the CNN due to the lack of the globally measured data. Accordingly, to train their parameters globally, federated learning strategy is exploited, known as an efficient learning strategy suitable to the multi-clients environment such as our system model shown in Figure 1.

Specifically, during the *t*th round of the training phase, each client receives the gradient of the model parameter $g_{t-1}$ from the server via the backhaul link. Then, by exploiting $g_{t-1}$ instead of $g_{t-1}^{\left(l\right)}$ in (3) the network function parameter can be updated as:

(6) $$\begin{array}{c} \theta_{t}^{\left(l\right)}\leftarrow \theta_{t-1}^{\left(l\right)}+g_{t-1}. \end{array}$$

We note that $g_{t-1}$ is the globally aggregated gradient computed at the server, which tends to minimize the loss function with respect to the data collected at all clients. Then, each client can compute its next local gradient $g_{t}^{\left(l\right)}$ such that the local loss function is minimized for the locally collected datasets $\Phi_{tr}^{\left(l\right)}$. Then, the locally updated gradient vector is reported to the server via the backhaul link. The server can then aggregate the local gradient vector to get $g_{t}$ as:

(7) $$\begin{array}{c} g_{t}=f_{g}\left(g_{t}^{\left(l\right)},\;l=1,\ldots ,L\right), \end{array}$$

where the function $f_{g}\left(\right)$ represents the gradient aggregation function. In [20], the FederatedAveraging technique (i.e., equal weight combining) is proposed which is given as:

(8) $$g_{t}=\frac{1}{L}\sum_{l=1}^{L}g_{t}^{\left(l\right)}.$$

The aggregated gradient $g_{t}$ is again broadcast to the multi-clients and exploited to update the neural network model at each client. The above described steps are repeated for a given number of rounds, *T*.

At the beginning of the training phase, the server needs to initialize the global model parameters and, throughout the paper, the parameters are initialized based on He normal weight initialization method [21], which is advantageous when used with ReLU activation function. Based on the above description, generalized federated learning process is summarized in Algorithm 1.
**Algorithm 1.** Generalized federated learning train process.  1:Initialize $\theta_{0}$ based on He normal weight initialization method  2:$g_{0}\leftarrow 0$  3:**for**t←1 to *T* **do**  4:      (Clients) $\theta_{t}^{\left(l\right)}\leftarrow \theta_{t-1}^{\left(l\right)}+g_{t-1}$  5:      (Clients) Update $g_{t}^{\left(l\right)}$ from the datasets $\Phi_{tr}^{\left(l\right)}$  6:      (Clients) Report $g_{t}^{\left(l\right)}$ to the server via the backhaul link  7:      (Server) $g_{t}\leftarrow f_{g}\left(g_{t}^{\left(l\right)},\;l=1,\ldots ,L\right)$ as in (7)  8:      (Server) Broadcast $g_{t}$ to multi-clients  9:**end for**

Differently from the centralized learning, the datasets collected by each client are not necessarily reported to the main server in Algorithm 1. We note that, in many cases, data sharing is not free from security, regulatory and privacy issues [8]. We also note that the communication cost for the centralized learning depends on the number/size of the collected data [22,23]. In contrast, the communication cost for the federated learning is independent with the data size, but depends on the CNN architecture (specifically, the number of weights in the CNN).

## 4. Gradient Updating and Adaptive Power Allocation Strategies for the Federated Learning over Wireless Backhaul

In line 6 of Algorithm 1, multi-clients should report their local gradient vectors $g_{t}^{\left(l\right)}$ through the backhaul link with *B* channel resources at each round. Specifically, each client should design the transmit signal $x_{l,t}$ to transmit $g_{t}^{\left(l\right)}$ in (5). In addition, the server should estimate ${\hat{g}}_{t}^{\left(l\right)}$ from the received signal $y_{t}$ in (5).

### 4.1. Linear Gradient Estimation for Federated Learning over Wireless Backhaul

To avoid the inter-channel interference over the wireless backhaul link, conventional orthogonal multiple access method with linear precoding is considered in which the wireless resource blocks are orthogonally allocated to each client. Specifically, by letting $\bar{B}=\frac{B}{L}$, which is assumed to be an integer, $x_{l}$ can be given as

(9) $$\begin{array}{c} x_{l,t}=\left[\begin{array}{c} 0_{\bar{B}\left(l-1\right)\times \bar{B}}\\ I_{\bar{B}}\\ 0_{\left(B-\bar{B}l\right)\times \bar{B}} \end{array}\right]\Psi_{\bar{B}\times \bar{P}}{\bar{g}}_{t}^{\left(l\right)}, \end{array}$$

where $\Psi_{\bar{B}\times \bar{P}}$ is a predefined pseudo-random matrix satisfying the restricted isometry property (RIP) condition [24] and unitary such as:

(10) $$\begin{array}{c} \Psi_{\bar{B}\times \bar{P}}^{H}\Psi_{\bar{B}\times \bar{P}}=\frac{1}{∥{\bar{g}}_{t}^{\left(l\right)}{∥}^{2}}I_{\bar{P}}. \end{array}$$

Note that $g_{t}^{\left(l\right)}\left(\in R^{P\times 1}\right)$ in (3) is split into multiple $\bar{P}$ dimensional vectors, ${\bar{g}}_{t}^{\left(l\right)}$ and each split vector is transmitted through $\bar{B}$ wireless resources.

Then, (5) can be rewritten as:

(11) $${\bar{y}}_{l,t}={\bar{H}}_{l,t}\Psi_{\bar{B}\times \bar{P}}{\bar{g}}_{t}^{\left(l\right)}+{\bar{n}}_{t},$$

where ${\bar{H}}_{l,t}=diag\left\{h_{l,t}\left[B\left(l-1\right)+1\right],h_{l,t}\left[B\left(l-1\right)+2\right],\ldots ,h_{l,t}\left[Bl\right]\right\}$ and

$${\bar{y}}_{l,t}=\left[\begin{array}{c} y_{t}\left[\bar{B}\left(l-1\right)+1\right]\\ \vdots \\ y_{t}\left[l\bar{B}\right] \end{array}\right],\;{\bar{n}}_{t}=\left[\begin{array}{c} n_{t}\left[\bar{B}\left(l-1\right)+1\right]\\ \vdots \\ n_{t}\left[l\bar{B}\right] \end{array}\right].$$

When $\bar{B}\geq \bar{P}$, $g_{t}^{\left(l\right)}$ can be estimated from (11) by exploiting the linear estimation methods such as zero-forcing or MMSE estimation. That is, ZF estimate of $g_{t}^{\left(l\right)}$ can be given as:

(12) $${\hat{\bar{g}}}_{t}^{\left(l\right)}={\left({\bar{G}}_{l,t}^{H}{\bar{G}}_{l,t}\right)}^{-1}{\bar{G}}_{l,t}^{H}{\bar{y}}_{l,t},$$

where ${\bar{G}}_{l,t}={\bar{H}}_{l,t}\Psi_{\bar{B}\times \bar{P}}$. When $\bar{B}<\bar{P}$ and $g_{t}^{\left(l\right)}$ is sparse, compressive sensing approach such as basis pursuit or orthogonal matching pursuit algorithms [25,26] can be applied to estimate $g_{t}^{\left(l\right)}$.

### 4.2. Proposed Gradient Updating Method Using Maximal Ratio Combining and Thresholding

From (12), the server can estimate the gradient reported from the *l*th client, ${\hat{\bar{g}}}_{t}^{\left(l\right)}$. Note that, because the channel gain of the wireless backhaul link is varying over the round during the federated learning process. The ill-conditioned channel with small channel gain may increase the estimation error and distort the gradient information associated with the *l*th client. Accordingly, in what follows, we propose two gradient update methods based on the channel gain, ${\bar{H}}_{l,t}$.

#### 4.2.1. Gradient Update by Maximum Ratio Combining

Note that the estimate of $g_{t}^{\left(l\right)}$ is more reliable for larger channel gain. To see this, by considering a simple case with $\bar{B}=\bar{P}$, we can rewrite (12) as:

(13) $$\begin{array}{ccc} {\hat{\bar{g}}}_{t}^{\left(l\right)} & = & \Psi_{\bar{B}\times \bar{P}}^{-1}{\left({\bar{H}}_{l,t}^{H}{\bar{H}}_{l,t}\right)}^{-1}{\bar{H}}_{l,t}^{H}{\bar{y}}_{l,t}\\ & = & {\bar{g}}_{t}^{\left(l\right)}+\Psi_{\bar{B}\times \bar{P}}^{-1}{\left({\bar{H}}_{l,t}^{H}{\bar{H}}_{l,t}\right)}^{-1}{\bar{H}}_{l,t}^{H}{\bar{n}}_{t}. \end{array}$$

  Accordingly, the mean squared estimation error is proportional to $\frac{\sigma_{n}^{2}}{∥{\bar{H}}_{l,t}{∥}_{F}^{2}}$. Equivalently, the effective SNR can be given as $\frac{∥{\bar{H}}_{l,t}{\bar{g}}_{t}^{\left(l\right)}{∥}_{F}^{2}}{\sigma_{n}^{2}}$. Therefore, when updating the aggregated gradient at the server from $g_{t}^{\left(l\right)}$, l=1,…,L, instead of (8), we can exploit the weighted sum of $g_{t}^{\left(l\right)}$ as

(14) $$g_{t}=\sum_{l=1}^{L}w_{t}^{\left(l\right)}{\hat{\bar{g}}}_{t}^{\left(l\right)},$$

where the weight $w_{t}^{\left(l\right)}$ that maximizes the effective output SNR can be derived as:

(15) $$w_{t}^{\left(l\right)}=\frac{∥{\bar{H}}_{l,t}{∥}_{F}^{2}}{\sum_{l=1}^{L}{∥{\bar{H}}_{l,t}∥}_{F}^{2}},\;$$

which is denoted as the maximum ratio combining (MRC) weights and allows the gradient vector that has undergone a better channel to contribute more to the aggregated gradient at the server. This is because it is more reliable and less-distorted through the wireless backhaul link, as observed from (13). To the best of our knowledge, the gradient update strategy by channel-based MRC in federated learning system with wireless backhaul has not been considered before.

#### 4.2.2. Binary Gradient Update by Thresholding

When the backhaul links for all clients have small channel gain simultaneously, the server may receive severely distorted gradient vectors even though it exploits the MRC strategy, such as (14). Accordingly, we propose a method in which the round associated with all channel having small channel gains is excluded from federated learning. Specifically, if $\sum_{l=1}^{L}{∥{\bar{H}}_{l,t}∥}_{F}^{2}<\epsilon$, the associated gradient is not updated at the server, where ϵ is a pre-defined constant. Based on the above description, the proposed federated learning process is summarized in Algorithm 2.
**Algorithm 2.** Proposed federated learning train process.  1:Initialize $\theta_{0}$ based on He normal weight initialization method  2:$g_{0}\leftarrow 0$  3:**for**t←1 to *T* **do**  4:      (Clients) $\theta_{t}^{\left(l\right)}\leftarrow \theta_{t-1}^{\left(l\right)}+g_{t-1}$  5:      (Clients) Update $g_{t}^{\left(l\right)}$ from the datasets $\Phi_{tr}^{\left(l\right)}$  6:      (Clients) Report $g_{t}^{\left(l\right)}$ to the server via the backhaul link  7:      **if** $\sum_{l=1}^{L}{∥{\bar{H}}_{l,t}∥}_{F}^{2}<\epsilon$ **then**  8:         (Server) $g_{t}\leftarrow \sum_{l=1}^{L}w_{t}^{\left(l\right)}{\hat{\bar{g}}}_{t}^{\left(l\right)}$ as in (14)  9:      **else**  10:         (Server) $g_{t}\leftarrow g_{t-1}$  11:      **end if**  12:      (Server) Broadcast $g_{t}$ to multi-clients  13:**end for**

### 4.3. Adaptive Power Allocation Strategy Based on the Gradient Information

When the transmission power of each client is limited, rather than allocating power equally to all channel resources (equivalently, slots), it is effective to allocate more power on the channel slots carrying specific important information. Note that, when training a deep learning model, the gradient elements with large values imply the large change of weight to decrease the loss function. Accordingly, because $g_{t}^{\left(l\right)}\left(\in R^{P\times 1}\right)$ in (3) is split into multiple $\bar{P}$ dimensional vectors, ${\bar{g}}_{t}^{\left(l\right)}$ in (9), each client allocate its transmit power proportionally to the magnitude of ${\bar{g}}^{\left(l\right)}$ in our proposed power allocation strategy. Assuming that $\bar{N}=P/\bar{P}$ is an integer and then, the number of multiple split vectors is given as $\bar{N}$. The adaptive power allocation strategy can be accomplished by setting:

(16) $$\Psi_{\bar{B}\times \bar{P}}^{H}\Psi_{\bar{B}\times \bar{P}}=\frac{\bar{N}}{∥g_{t}^{\left(l\right)}{∥}^{2}}I_{\bar{P}}.$$

We note that the constraint of (10) allows the equal power to be used when transmitting the split vector ${\bar{g}}_{t}^{\left(l\right)}$, while the constraint of (16) allows the power to be used in proportion to the magnitude of ${\bar{g}}_{t}^{\left(l\right)}$ at each transmission, exhibiting the same total transmit power as in (10). In addition, the power allocation as (16) has not been considered in the conventional federated learning methods over wireless channels.

## 5. Experiment Results

To see the validation of the proposed federated learning train strategy discussed in Section 4, we develop the CNN module for handwriting character recognition having the architecture in Figure 2. Specifically, the CNN module has three two-dimensional convolutional layers and the values for the hyperparameters exploited in the computer simulations are summarized in Table 1. Then, the number of elements in the gradient vector $g^{\left(l\right)}$ is given as $5.26\times 10^{4}$. The CNN module is shared by three clients connected to the server over the wireless channel. Throughout the simulations, we exploit the handwriting MNIST dataset where $N_{width}=N_{height}=28$. In addition, three clients are considered and the received SNR at the server is defined as:

(17) $$\begin{array}{c} SNR_{rec}=\frac{\sum_{l=1}^{L}\sigma_{h_{l}}^{2}}{L\sigma_{n}^{2}}, \end{array}$$

where $\sigma_{n}^{2}$ is the variance of the AWGN. In addition, we split the gradient vector into multiple vectors having 128 elements (i.e., $\bar{P}=128$ in (9)).

**Figure 3:**
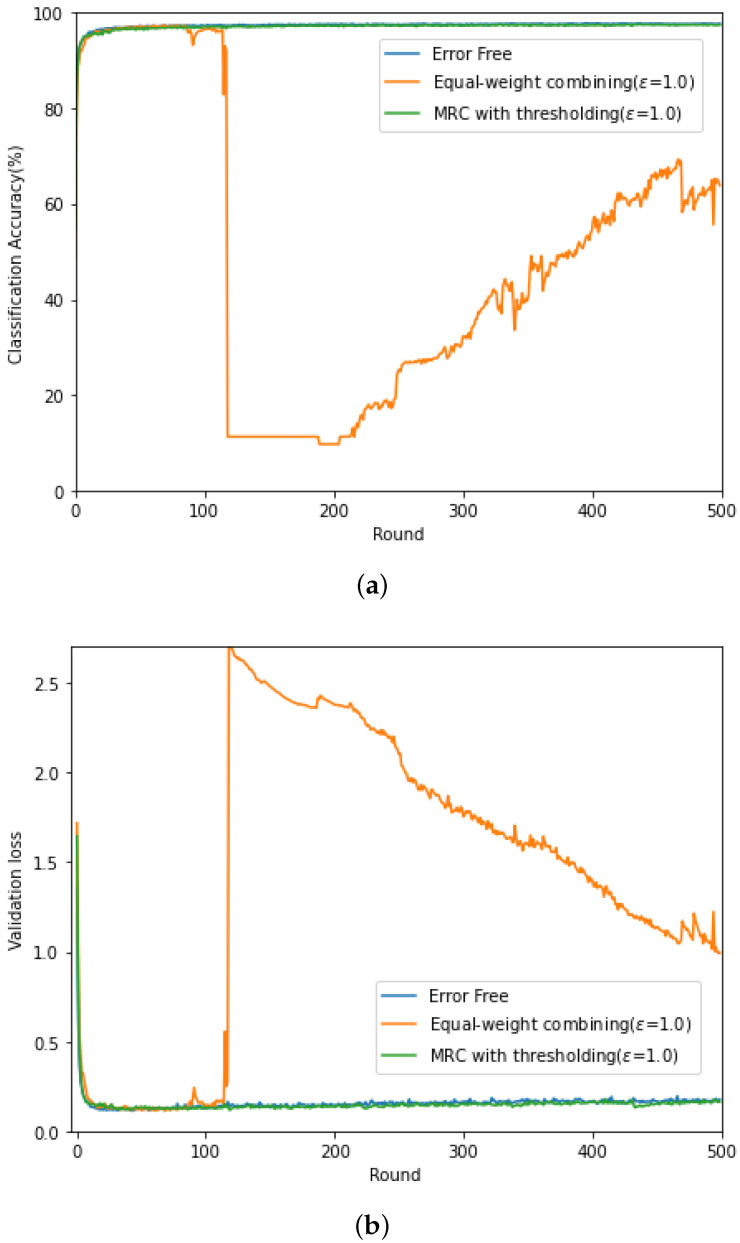
(**a**) Classification accuracy and (**b**) CE loss curves at $SNR_{rec}=15$ dB.

**Figure 4:**
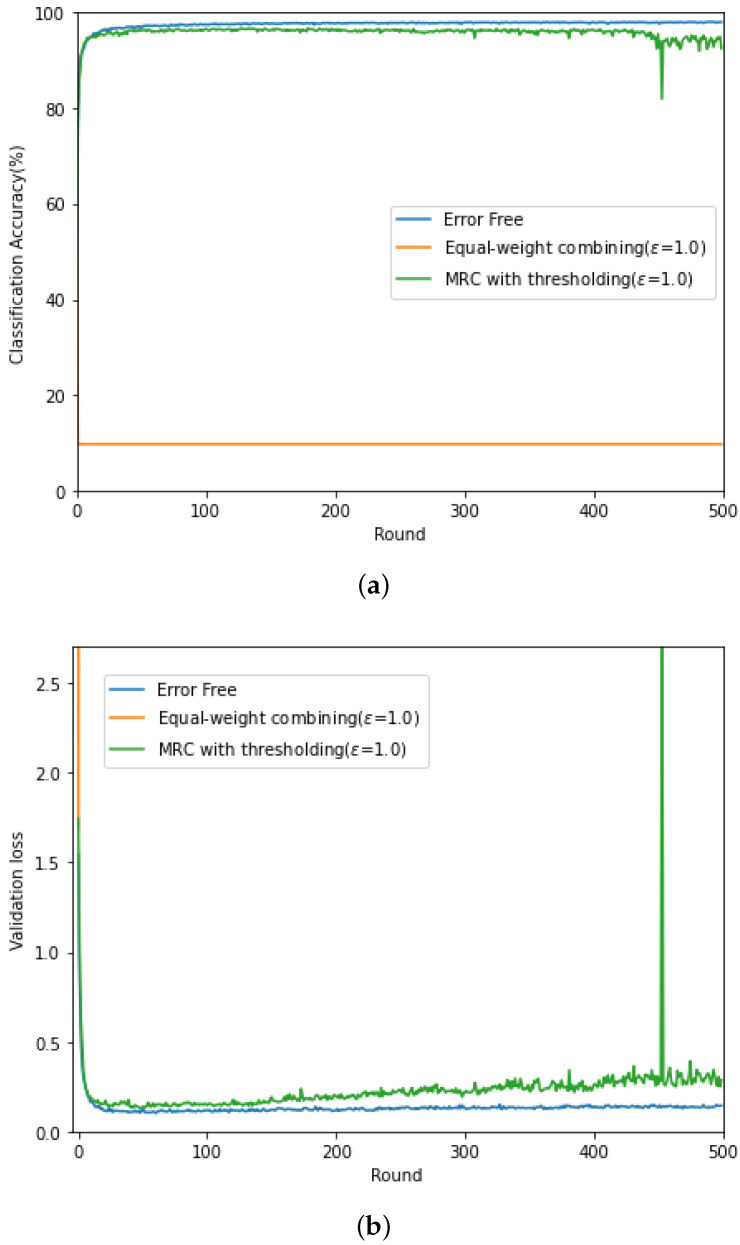
(**a**) Classification accuracy and (**b**) CE loss curves at $SNR_{rec}=-10$ dB.

In Figure 3 (respectively, Figure 4), we evaluate the classification accuracy and CE loss of the conventional gradient updating method based on the equal-weight combining and the proposed updating method based on MRC, discussed in Section 4.2 for high SNR ($SNR_{rec}=15$ dB) (respectively, low SNR ($SNR_{rec}=-10$ dB)). For comparison purposes, the performance of the federated learning with error-free backhaul link is also evaluated. Here, the channel gain of each client is set as $\sigma_{h_{l}}^{2}=\left\{0.3,1.0,3.0\right\}$ and the threshold level in given as ϵ=1.0, and this value was experimentally determined. For the local training of the commonly shared CNN module, ADAM optimizer is adopted [27] at each client with a fixed learning rate, η=0.001.

From Figure 3, when the backhaul link is perfect and noise free, the classification accuracy increases in proportion to the rounds and the accuracy up to 0.97 can be achieved. In contrast, due to the channel fading and noise in the wireless backhaul link, training does not proceed stably when the conventional equal-weight combining is exploited. In Round 120, there is a sharp increase at the loss curve from 0.28 to 2.75, resulting in the decrease in the accuracy from 0.92 to 0.11. In contrast, the performance of the proposed updating method based on MRC in Section 4.2 exhibits a similar performance to that with the perfect backhaul link. In Figure 4, it can be found that, for low SNR, the classification accuracy of the equal-weight combining is not improved as the rounds increases and is below 0.15. In addition, the associated CE loss goes to infinity. At low SNR, it is difficult to recover the distortion caused over the wireless backhaul link when transmitting the gradient for model update. Especially, when there is channel distortion, the equal-weight combining does not reflect the received SNR in the gradient update and fails to train the distributed CNN modules. Interestingly, the updating method based on MRC and thresholding shows unstable peak in the CE loss, but it can avoid the CE loss divergence and improve the classification accuracy as the learning round increases.

**Figure 5:**
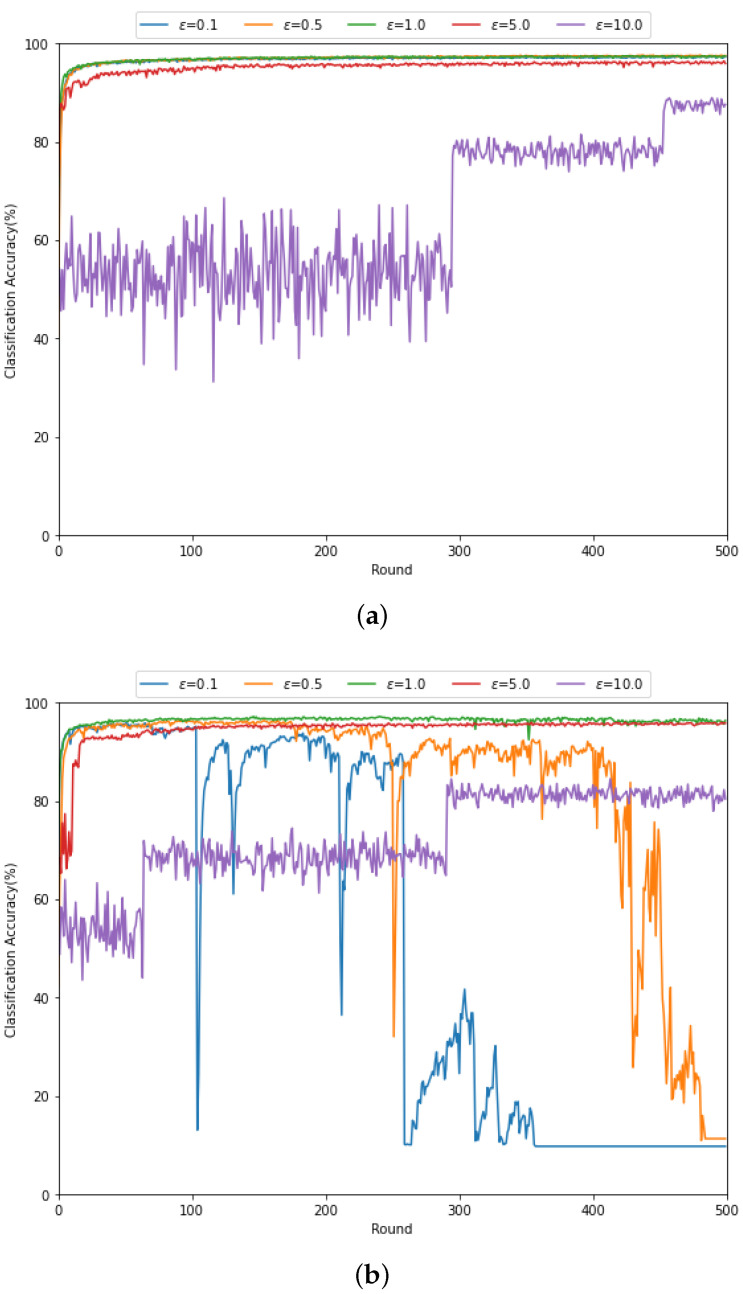
Classification accuracy according to various threshold levels when the updating method with MRC and thresholding in Section 4.2.2 are exploited for (**a**) $SNR_{rec}=15$ dB and (**b**) $SNR_{rec}=-10$ dB.

In Figure 5, we evaluate the classification accuracy for various threshold levels ϵ with (a) $SNR_{rec}=15$ dB and (b) $SNR_{rec}=-10$ dB when the updating method with MRC and thresholding in Section 4.2.2 is exploited. From Figure 5a, at high SNR, the federated learning can be well operated through the gradient updating method with MRC and thresholding, regardless of the threshold levels. However, for ϵ=10.0, the accuracy does not effectively increase as the learning round increases. That is, for a larger threshold level, more local gradient vectors transferred through the wireless channel can be discarded. In Figure 5b, it can be found that the classification performance is more sensitive to the threshold level at low SNR compared to the high SNR case. Specifically, as ϵ is larger, the federated learning is performed stably. This is also because the gradient update vector containing the amplified noise due to small channel gain can be discarded for large ϵ. We note that the large ϵ may incur the gradient updating delay, which leads the trade-off between the federated learning performance and the learning delay.

**Figure 6:**
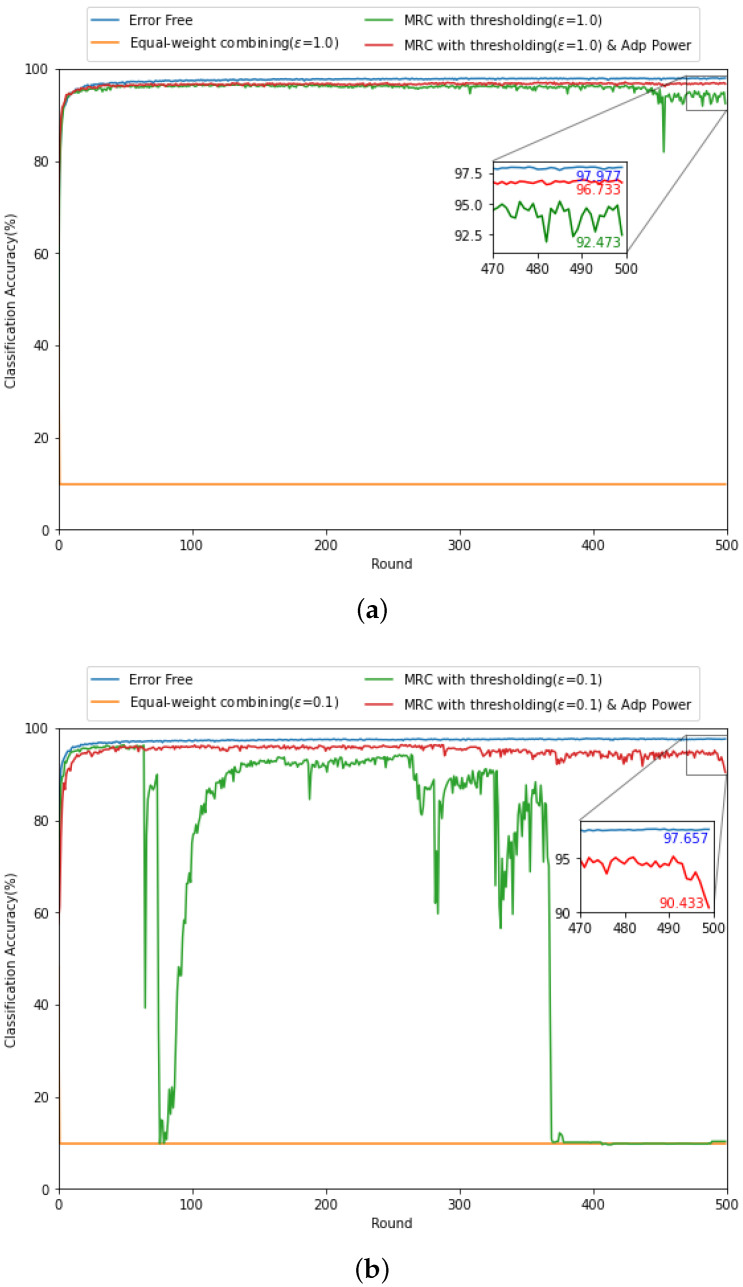
Comparison of classification accuracy with (**a**) ϵ=1.0 and (**b**) ϵ=0.1 for $SNR_{rec}=-10$ dB.

In Figure 6, to validate the adaptive power allocation strategy in Section 4.3, we evaluate the classification accuracy of various gradient updating methods with/without the adaptive power allocation strategy when the received SNR is low with different threshold levels (i.e., (a) ϵ=1.0 and (b) ϵ=0.1). It can be found that the accuracy of the MRC based gradient updating method with ϵ=1.0 in Figure 6a is more stable compared to that with ϵ=0.1 in Figure 6b, which coincides with the observation in Figure 5. Interestingly, by exploiting the adaptive power allocation strategy jointly with the MRC based gradient updating method in Figure 6a, the accuracy can be improved by 96.7% and it is comparable to the performance with error-free backhaul link. In addition, from Figure 6b, the adaptive power allocation strategy drastically stabilizes the federated learning performance during the learning process over wireless channel even for small ϵ=0.1. Accordingly, the adaptive power allocation strategy improves the trade-off between the federated learning performance and the learning delay due to the threshold level discussed in Figure 5.

In Table 2 and Table 3, the confusion matrices for the test dataset are evaluated after the federated learning is completed, where the proposed gradient updating method (Table 2) and the conventional updating method (Table 3) are, respectively, exploited. From Table 2, the proposed gradient updating method shows the classification accuracy of 0.9 or more for all labels. However, from Table 3, the CNN module trained through the conventional gradient updating method over wireless channel misclassifies most test data with specific labels.

## 6. Conclusions

In this paper, efficient gradient updating strategies are developed for federated learning when distributed clients are connected to the server via a wireless backhaul link. That is, a common CNN module is shared for all the distributed clients and it is trained through the federated learning over wireless backhaul connected to the main server. During the training phase, local gradients need to be transferred from the distributed clients to the server over a wireless noisy backhaul link. To overcome the distortion due to wireless channel fading, an effective SNR maximizing gradient updating method is proposed, in which the gradients are combined such that the effective SNR is maximized at the server. In addition, when the backhaul links for all clients have small channel gain simultaneously, the server may have severely distorted gradient vectors. Accordingly, we propose a binary gradient updating strategy based on thresholding in which the round associated with all channels having small channel gains is excluded from federated learning, which results in the trade-off between the federated learning performance and the learning delay. Due to the channel fading and noise in the wireless backhaul link, training does not proceed stably with the conventional equal-weight combining especially at low SNR. In contrast, the updating method based on MRC and thresholding improves the classification accuracy as the learning round increases by avoiding the CE loss divergence. Finally, we also propose an adaptive power allocation method, in which each client allocates its transmit power proportionally to the magnitude of the gradient information. Note that the gradient elements with large values imply the large change of weight to decrease the loss function. Through the computer simulations, it is confirmed that the adaptive power allocation strategy can improve the trade-off between the federated learning performance and the learning delay due to the threshold level.

## Figures and Tables

**Table 1:** The values for the hyperparameters of the CNN module for handwriting character recognition.

	Values
The number of layers	3
The number of filters at each layer	3
Filter size (The 1st layer), $K_{f11}\times K_{f12}$	(1×16)
Filter size (The 2nd layer), $K_{f21}\times K_{f22}$	(16×32)
Filter size (The 3rd layer), $K_{f31}\times K_{f32}$	(32×64)
Optimizer	ADAM optimizer [27]
Learning rate, η	0.001

**Table 2:** Confusion matrix for hand writing character recognition of the proposed gradient updating method.

Predicted	True Label									
Label	0	1	2	3	4	5	6	7	8	9
0	**0.984**	0.001	0.020	0.004	0.005	0.006	0.008	0.003	0.022	0.007
1	0	**0.979**	0.002	0	0.005	0.001	0.002	0.002	0.001	0.001
2	0	0.004	**0.943**	0.005	0	0	0	0.015	0.004	0
3	0	0.001	0.006	**0.963**	0	0.006	0	0	0.007	0.007
4	0	0.001	0.001	0	**0.929**	0	0.002	0.001	0.005	0.004
5	0	0.001	0	0.019	0.001	**0.955**	0.005	0.001	0.003	0
6	0.010	0.005	0.003	0	0.016	0.015	**0.980**	0	0.007	0.001
7	0.001	0.001	0.016	0.007	0.001	0.003	0	**0.949**	0.007	0.016
8	0	0.007	0.009	0.002	0.001	0.011	0.001	0.003	**0.915**	0.006
9	0.005	0	0.001	0	0.042	0.003	0.001	0.026	0.029	**0.961**

**Table 3:** Confusion matrix for hand writing character recognition of equal-weight combining based gradient updating method.

Predicted	True Label									
Label	0	1	2	3	4	5	6	7	8	9
0	**0.010**	0.004	0.012	0.004	0.011	0.026	0.010	0.002	0.005	0.001
1	0	**0**	0	0	0	0	0	0	0	0
2	0.977	0.875	**0.961**	0.877	0.932	0.777	0.971	0.842	0.919	0.943
3	0	0	0	**0**	0	0	0	0	0	0
4	0.009	0.085	0.026	0.113	**0.039**	0.172	0.017	0.126	0.059	0.036
5	0	0	0	0	0	**0**	0	0	0	0
6	0	0	0	0	0	0	**0**	0	0	0
7	0	0.025	0.001	0.002	0.016	0	0	**0.022**	0	0.020
8	0.004	0.011	0	0.004	0.002	0.026	0.002	0.007	**0.017**	0.001
9	0	0.001	0	0	0	0	0	0	0	**0**

## Data Availability

Not applicable.
